# Understanding Transgender and Gender-Diverse Youth’s Experiences Receiving Care via Telemedicine: Qualitative Interview Study

**DOI:** 10.2196/42378

**Published:** 2023-02-14

**Authors:** Nicole F Kahn, Yomna H Anan, Kevin M Bocek, Dimitri A Christakis, Laura P Richardson, Wanda Pratt, Gina M Sequeira

**Affiliations:** 1 Department of Pediatrics University of Washington School of Medicine Seattle, WA United States; 2 Division of Adolescent Medicine Seattle Children's Hospital Seattle, WA United States; 3 Center for Child Health, Behavior and Development Seattle Children's Research Institute Seattle, WA United States; 4 The Information School University of Washington Seattle, WA United States

**Keywords:** transgender and gender diverse youth, adolescent, telemedicine, gender-affirming care, qualitative methods, COVID-19, pandemic, youth, gender, care, technical, implementation, transgender, telemedicine, gender diverse, complexity

## Abstract

**Background:**

Access to virtual care has increased since the beginning of the COVID-19 pandemic, yet little is known about transgender and gender-diverse (TGD) youth’s experiences and perspectives on receiving care via telemedicine.

**Objective:**

The purpose of this study was to explore these experiences to (1) inform necessary changes to the provision of pediatric gender-affirming care and (2) help providers and health systems determine if and how telemedicine should be made available post pandemic.

**Methods:**

Youth (aged 14-17 years) who completed a telemedicine visit in the Seattle Children’s Gender Clinic were invited to participate in a semistructured interview exploring perceived advantages or disadvantages of telemedicine and preferred visit modalities. Interview transcriptions were analyzed by 2 research team members using an inductive thematic analysis framework.

**Results:**

A total of 15 TGD youth completed an interview. Commonly cited advantages of telemedicine were convenience and comfort with having visits in their own environments. Reported disadvantages included technical issues, discomfort with the impersonal nature, lack of familiarity with the platform, and privacy concerns. Overall, slightly more youth preferred in-person visits over telemedicine, referencing both specific characteristics of the clinical visit (ie, initial vs return and complexity) and proximity to the clinic as reasons for this preference. Although a plurality of TGD youth preferred in-person visits, they also recognized the value of telemedicine and the impact it may have in facilitating access to care.

**Conclusions:**

Given the variations in needs and visit complexity, our study supports the provision of both in-person and telemedicine modalities as options for pediatric gender-affirming care.

## Introduction

Telemedicine provides a means for 2-way, real-time, synchronous communication between a health care provider and a patient, who are not in the same physical location, using audio or video technology [1]. Although telemedicine has been available for a number of years, it has primarily been used to support populations who experience geographic barriers to specialty health care (eg, rural settings and health professional shortage areas) [1]. However, the COVID-19 pandemic has recently forced health care systems to rapidly implement or scale up the availability of telemedicine visits to continue serving patients, resulting in increased focus on optimizing these platforms for broader use [2,3].

Even before the pandemic, work was in progress to expand telemedicine for the provision of subspecialty care to other vulnerable populations, including children and youth [4]. This was also true for transgender and gender-diverse (TGD) youth seeking access to gender-affirming care, which may include social or psychological supports and medical care that affirms an individual’s gender identity. Telemedicine represented an important mode of care delivery for TGD youth, given that many experience substantial barriers to receiving gender-affirming care due to the limited number of clinics across the United States that deliver this care and the fact that few providers outside of these clinics have received formal training in this area [5-7]. However, few guidelines were in place for the provision of these services [3], and little research had been conducted regarding youth’s needs and preferences with this modality of care delivery [8,9].

More recent research during the pandemic with a nationally representative sample of youth has shown that young people see value in telemedicine for minor concerns or follow-up care, but that most still prefer in-person visits [10]. Similarly, research conducted before the pandemic showed that just under half (47%) of TGD youth were interested in telemedicine, but they, too, preferred to use this modality for follow-up care [9,11,12]. However, telemedicine interest was especially high among TGD youth who had less parental support for their identities [11]. Such unique experiences illustrate the importance of understanding diverse patient perspectives as health care systems make decisions about whether and how to provide telemedicine services moving forward. As such, now that telemedicine services are more widespread, more research is needed to understand TGD youth’s perspectives on receiving gender-affirming care via telemedicine and whether this could be a way to improve access and help overcome the unique barriers to care faced by this population [5,6,13].

Therefore, the purpose of this study was to further explore TGD youth’s experiences receiving gender-affirming care via telemedicine, with the broader goals of (1) informing necessary changes to the provision of pediatric gender-affirming care and (2) helping gender-affirming care providers and health systems determine if and how this modality should continue to be made available following the COVID-19 pandemic.

## Methods

### Participants and Recruitment

Youth aged 14-17 years who completed a telemedicine visit with a Seattle Children’s Gender Clinic physician or nurse practitioner to discuss or receive gender-affirming medical care within the last 6 months were invited to complete a screening survey, and if eligible, to participate in a semistructured, one-on-one Zoom interview. For the purpose of this study, the term “telemedicine” was defined as any visit that occurred using real-time video and audio technology from a location outside of the clinic.

For initial recruitment, members of the research team reviewed electronic medical records to identify patients who met initial inclusion criteria to contact by email or during an in-person visit to the clinic. All prospective participants were directed to a Research Electronic Data Capture (REDCap) [14] screening survey to determine eligibility and to provide additional information about the study. Eligible participants were then contacted by a member of the research team to schedule an interview over Zoom [15].

### Ethics Approval

Due to the low-risk nature of this study and to avoid excluding participants who may not have disclosed their gender identity to a parent, a waiver of parental consent was granted by the Seattle Children’s Hospital Institutional Review Board (STUDY00002873), and all participants provided verbal assent to participate prior to the start of the interview. Each interview participant received a US $20 Amazon e-gift card. All study procedures were approved by the Seattle Children’s Institutional Review Board.

### Measures

#### Screening Survey

Demographic information regarding age, gender identity, ethnicity, and race were self-reported on the REDCap screening survey.

For gender identity, respondents could select all that applied from the following: transmale or transmasculine, transfemale or transfeminine, nonbinary, genderqueer, genderfluid, gender questioning, gender nonconforming, agender, demigender, gender variant, androgyne, two spirit (or other identity of indigenous origin), cisgender male, cisgender female, and other.

Respondents were also asked about travel time to the clinic, how supportive their most supportive parent or legal guardian was of their transition on a scale from 1 to 10, and whether a parent had participated in their telemedicine visit with the gender clinic.

#### Interview

The first part of the semistructured interview included open-ended questions related to participants’ experiences receiving gender-affirming care via telemedicine. The second half of the interview focused on their attitudes toward receiving gender-affirming care in the primary care setting. This paper focuses on the first half of the interview, which asked the following open-ended questions:

- “How did you feel about doing a gender clinic visit using telemedicine?”
- “Were there particular aspects of the telemedicine visit you liked or didn't like? Why?”
- “If you had the option of doing a visit over telemedicine vs in-person, what would you choose and why?”

Interview questions were reviewed by members of a TGD youth advisory board for clarity and applicability prior to implementation.

#### Analyses

Interview transcripts were automatically generated by the Zoom computer program [15] and reviewed by members of the research team for clarity and accuracy. Transcripts were then analyzed by 2 members of the research team using an inductive thematic analysis framework [16] and Dedoose qualitative analysis software (SocioCultural Research Consultants) [17]. Specifically, 2 members of the research team (“coders”) used an initial codebook that was generated collaboratively with TGD youth, parent, and primary care provider stakeholders to analyze the data. The coders then met weekly to discuss disagreements and to add codes iteratively as other key themes emerged from the interviews until thematic saturation was achieved. The final results were triangulated with members of a TGD youth advisory board who were provided with a list of themes and examples to ensure they agreed with the quote categorizations.

## Results

### Participant Characteristics

Of the 45 TGD youth who completed the screening survey, 29 (64%) were deemed eligible and invited for an interview, of which 15 (52%) agreed to participate (Table 1). The mean age of interview participants was 15.7 years (SD 1.1). The majority (n=11, 73%) identified as transmale or transmasculine, and one-third (n=5, 33%) selected more than one gender identity (range: 1-8). Most of the interviewed youth (n=12, 80%) completed their first gender-clinic visit in person, while 3 (20%) youth had completed their first gender-clinic visit via telemedicine. Nearly all of the interviewed TGD youth lived within an hour from the clinic (n=14, 93%). Overall, youth indicated high levels of support for their transition from a parent (mean 8.5, SD 1.9), and 12 (80%) youth had a parent join for a telemedicine visit. Interviews ranged in length from 20 to 45 minutes.

**Table 1:** Demographic characteristics of screening survey and interview participants.

Variables		Screened (n=45)	Interviewed (n=15)
Age (years), mean (SD)		16.2 (1.2)	15.7 (1.1)
**Gender identity^a^, n (%)**			
	Transmale or male	30 (62.5)	11 (73.3)
	Transfemale or female	11 (22.9)	3 (20.0)
	Nonbinary	14 (29.2)	6 (40.0)
	Genderqueer	5 (10.4)	3 (20.0)
	Genderfluid	2 (4.2)	2 (13.3)
	Gender questioning	0 (0.0)	0 (0.0)
	Gender nonconforming	3 (6.3)	3 (20.0)
	Agender	4 (8.3)	1 (6.7)
	Demigender	4 (8.3)	2 (13.3)
	Gender variant	1 (2.1)	1 (6.7)
	Androgyne	0 (0.0)	0 (0.0)
	Two spirit (or other identity of indigenous origin)	1 (2.1)	0 (0.0)
	Cismale	0 (0.0)	0 (0.0)
	Cisfemale	0 (0.0)	0 (0.0)
	Bigender	2 (4.2)	1 (6.7)
	>1 gender identity (range: 1-8)	15 (33.3)	5 (33.3)
**Ethnicity or race^a^, n (%)**			
	Hispanic	9 (18.8)	2 (13.3)
	American Indian or Alaska Native	2 (4.2)	0 (0.0)
	Asian	5 (10.4)	2 (13.3)
	Black or African American	3 (6.3)	1 (6.7)
	Native Hawaiian or Pacific Islander	2 (4.2)	1 (6.7)
	White	37 (77.1)	13 (86.7)
**Distance to clinic, n (%)**			
	≤30 minutes	10 (20.8)	5 (33.3)
	31 minutes to 1 hour	25 (52.1)	9 (60.0)
	1 to 2 hours	4 (8.3)	0 (0.0)
	2 to 3 hours	0 (0.0)	0 (0.0)
	3 to 4 hours	2 (4.2)	1 (6.7)
	>4 hours	2 (4.2)	0 (0.0)
**First visit, n (%)**			
	In person	29 (60.4)	12 (80.0)
	Telemedicine	14 (29.2)	3 (20.0)
Parental support for transition (mean; 1-10)		8.2 (2.1)	8.5 (1.9)
Parent at telemedicine visit, n (%)		38 (79.2)	12 (80.0)

^a^Youth could select more than one gender identity and ethnicity or race.

### Advantages and Disadvantages of Telemedicine Modality for Gender-Affirming Care

Key themes of the TGD youth’s responses regarding the aspects that they liked and disliked about telemedicine with representative quotes are shown in Table 2.

**Table 2:** Advantages and disadvantages of telemedicine for gender-affirming care (n=15).

Themes and subthemes			Quotations
**Advantages**			
	**Convenience (n=13, 87%)**		
		Travel time	*“I do like that it's more convenient. Like you just don't have to, like, drive out or get your parents to drive you out to a specific location.”* *“I especially appreciated that for very short check-in visits. That was, like, super easy to do via telemedicine. That would have taken up a big portion of my day if we'd had to drive down.”*
		Efficiency	*“There is also no wait time. I had some long waiting in the office times with the gender clinic. But with telemedicine, it’s just like, you log on and they're right there. So, it's very, very efficient.”* *“I think for like just checkups and stuff and general symptoms it's good for telehealth because you can just call and make it happen instead of having to drive over.”*
		User-friendly	*“It's very like easy to use the telemedicine videos like you can, like it's very like easy to learn like it's not really complicated like you can just, press on the Zoom link and then go on and then, if you have to put your password or your name you just click enter and then and it's super reliable too, so I like it.”* *“Telemedicine is easy, you can do it at your house, in your bed, you don't really have to do much, you just tap a few buttons.”*
	**Comfort (n=10, 67%)**		
		Own environment	*“Well, I mean it's nice to do visits from my house. It's like being in my home is a little bit more comforting because you know, when I go to [CLINIC NAME] I'm in a hospital.”* *“Um, for me, it's mostly the environment around me because when I'm actually in clinic it doesn't really have that same feeling that at home it does, cuz that’s home…it's home. But the clinic, it feels like there's a bunch of doctors and nurses and it just doesn't have that same comfort that it would be at home.”*
		Social anxiety	*“It might be scary you're going to in person, because they've never had to, cause gender is, like a really touchy subject and, like it can be a little bit uncomfortable sometimes, so I think having the screen in between can sometimes make it a little bit less scary.”* *“For me, especially back then, before I started hormones, I was a very socially anxious person. So, it’s always a little daunting going in and sitting face to face with somebody for an hour and you’re just looking at a doctor and I feel like through telemedicine it kind of broke that weird barrier there. Like I wasn't as anxious going into it and it was a really good introduction into my care there.”*
		Avoiding COVID-19	*“Personally, I like it just because like especially right now, because, like COVID and, like the delta variant and all that stuff going on, I don't feel super comfortable going in, even though I am vaccinated like fully but I do like in this current time, the distance because of the health precautions and stuff.”* *“especially with COVID it- it's it it's a lot safer”*
**Disadvantages**			
	**Technical issues (n=9, 60%)**		
		Bad connection	*“Occasionally it's annoying if like, connections are bad and it's hard to understand people, technology issues, that sort of thing. But that's about it. That's pretty much the only issues I’ve had.”* *“It's not really the telemedicine, like visit. It's more like the, like if you live somewhere like, where the Internet is weird like, sometimes the connection is like, unstable.”*
		Difficulty using platforms	*“I think, I think it's a bit difficult sometimes because of planning around it and about environment. Because it can be stressful to figure out how to set up something like Zoom or Teams if you haven't before.”* *“I don’t personally have any concerns but I know that some people might struggle with a public video call software via the platform that we use. It might be more comfortable for some people to be able to do theirs through something that can be assured as limited access and very secure. Like a [HOSPITAL NAME] run portal type thing.”*
	**Discomfort (n=8, 53%)**		
		Impersonal	*“There is kind of more of a personal aspect to sitting with somebody. You kind of get to be with them and it's more of a close connection you have with them.”* *“I just think sitting in a room with someone and seeing them right there kind of helps you get more of a feeling of ‘this is a real person.’ Because sometimes, looking at a screen, it's just ‘this is my computer.’ There’s just this barrier in between you and the other person.”*
		Unfamiliar	*“It's just a different environment and it changes how you feel about a certain area.”* *“I think at first it was very unfamiliar to me and kind of hard to adjust with talking to someone on a screen instead of in person. Especially about kind of personal things, but that wasn't an issue much as I got more used to doing that.”*
	**Privacy (n=7, 47%)**		
			*“Some of the things that I don't really like is because everybody's at home, I feel like people will overhear me because the walls aren’t that thick and, or like they'll come up to me and say like, ‘hey I heard you say this over your interview’ or like, ‘during your visit.’”* *“Like the contrast between being in person and being like, through Zoom - when you're in person you're in an office or you're in a room somewhere and it's just the two of you, and you know that it's private. But then when you're not, on their end it's private but also like, on your end, you can be anywhere.”*

#### Advantages

The 2 main themes that emerged regarding the advantages of telemedicine included *convenience* (n=13, 87%) and *comfort* (n=9, 60%). Regarding convenience, participants appreciated not having to commute to the clinic, the efficiency of their visit, and the user-friendliness of the web-based platform. Regarding comfort, TGD youth liked being in their own environment to conduct the visit, not having to deal with social anxiety surrounding their gender identity when visiting the clinic, and avoiding exposure to COVID-19.

#### Disadvantages

The most commonly reported disadvantage of conducting the visit over telemedicine was *technical issues* (n=9, 60%). Specifically, TGD youth cited unreliable internet connections and difficulty navigating a new or different platform to engage with their providers. Just over half of the TGD youth also reported *discomfort* (n=8, 53%) using telemedicine, indicating that these visits felt impersonal and were an unfamiliar way of engaging with their providers. Finally, participants frequently voiced concerns about *privacy* (n=7, 47%), explaining that they felt more likely to be overheard by family or others while connecting with their providers from home.

### Preferred Modalities for Receiving Gender-Affirming Care

A slight majority of the interviewed youth (n=7, 47%) indicated that they preferred in-person visits compared to telemedicine (n=5, 33%), while the remaining 3 (20%) youth said it would depend on certain characteristics of the visit. Interestingly, although most youth had a specific preference for either in-person or telemedicine modality, it became clear through further discussion that their preferred modality could vary based on certain characteristics of the visit (Table 3).

Specifically, youth indicated that their first visit to the gender clinic would be better done in person, but for return or follow-up visits, they preferred telemedicine. In fact, of the 5 youth who preferred telemedicine visits, 3 (60%) indicated wanting their first visit to be in person. Regarding the first visit, participants cited wanting to build rapport with their provider and have their initial conversations occur face-to-face. Participants who preferred telemedicine for follow-up or return visits most often indicated that this was due to the simplicity of the visit and the frequency with which they occur.

Another main theme was visit complexity. Participants suggested that complex visits, including those that involved procedures or major changes to their care, were better done in person. Conversely, participants indicated that less complex visits, including verbal check-ins with a provider, could be easily completed via telemedicine.

Finally, youth cited distance to the clinic as an important factor in deciding when to use telehealth services. Specifically, youth suggested that in-person visits were better for those who lived closer to the gender clinic, while telemedicine were better for those who lived further away or with other transportation barriers.

The following quote from a study participant illustrates this interplay between these characteristics:

My first visit with the gender clinic was definitely better in person, but many consecutive appointments after that, I would have absolutely done telemedicine. Because there was my first appointment, I was prescribed something out the gate and that's, you know, I would prefer that to be in person. But every subsequent appointment besides that wasn't talking about starting a new medication. It was definitely like, I’m here for 10 minutes and I’m out. All right, well goes back to convenience, where I don't really want to hear the same thing, I don't want to go drive half an hour and then hear the same thing that I heard last appointment then drive away…I guess it depends on the subject matter and the duration of the visit...But yeah, yeah it's not, it, I feel like, ironically, despite my stance on telemedicine, like, as in, like, it's probably my least preferred way of communicating medical ideals, it's, I also believe that it's the future of medicine, because it's so convenient.

**Table 3:** Reasons for in person versus telemedicine care.

Themes	Visit description and quotations	
	In-person	Telemedicine
Visit type	First visit *“I personally would prefer the first appointment to be in person, just so it's easier to connect.”* *“I think maybe go in person, just for like a big initial visit, and then after that I would have just been comfortable with telemedicine.”*	Return visit *“I think follow ups it really makes sense to do them virtually because you're gonna have so many of them.”* *“I honestly think that telemedicine makes a lot more sense than in person, especially for follow ups” *
Complexity	More complex visit *“If we were going to talk about doing something new or making a big change to the stuff I'm already doing, I think that's something that would be better done face-to-face.”* *“If they need to, you know, run tests, draw blood or whatever like that, then I would rather come in then, be in person, because I feel like that's harder to do virtually.”*	Less complex visit *“It kind of depends what the subject of the visit would be, but for the normal check-ins I do, I think telemedicine is preferable.”* *“I think it depends on what needs to be done at my visit, because for just an average ‘how are you doing on your hormones’ kind of talking thing, I would prefer telemedicine.” *
Distance	Closer proximity to clinic *“I don't think, there's no challenges or obstacles because like, we live like, really close to her like, well, to the hospital.”* *“I guess if you're closer to [CITY] and, and would, you would be, different reasons.”*	Further distance from clinic *“Personally, I thought it was a lot easier because I live a few hours away.”* *“I think time is a big part of it…Like, for me, I prefer telemedicine because it's a long time to drive.” *

## Discussion

### Principal Findings

The purpose of this study was to better understand TGD youth’s perspectives and preferences regarding how they receive gender-affirming care services in order to inform and improve future care delivery. Our results support those of past surveys and quantitative research, which have shown that youth prefer in-person visits to telemedicine [8-11]; however, our qualitative discussions regarding benefits and drawbacks of each modality further indicate that the choice is not so simple. Although a plurality of TGD youth in our study indicated a preference for in-person visits, they also recognized the value of telemedicine to themselves and others for less complex or follow-up visits and the impact it may have in facilitating access to care [12]. Given variations in needs and visit complexity, our results suggest that both in-person and telemedicine modalities should be options for the provision of gender-affirming care, and that patient needs and constraints should be considered as these services become more widely implemented.

Although the rapid implementation and scale-up of telemedicine options resulting from the COVID-19 pandemic have allowed health care systems to continue serving patients remotely, any such rapid change also brings growing pains [2,3]. Some of these may be remediable with time. For example, certain disadvantages cited by the youth in our study, including discomfort with the new modality and certain technical issues, may improve as patients become more familiar with the visit process and platforms used. Other disadvantages, such as feeling impersonal, may also be helped by additional provider training on building rapport virtually. However, there are some disadvantages, such as privacy issues, which may be more difficult to change given the different environments where youth engage in their telemedicine visits. National guidelines emphasize the importance of confidential health services for adolescents, which allow for private discussion of sensitive health topics and encourage adolescent responsibility for their own health care [18,19]. This may be even more important for TGD youth who are not open about their gender identity to all of the individuals with whom they live or those who have limited parental support [11,20]. Additional changes to virtual care provision (eg, room scans, use of headphones, asking whether the patient is alone or if they can be overheard at the start of the visit) may help to alleviate some of these privacy concerns, but further solutions to ensure access to confidential care via telemedicine are warranted [21].

### Strengths and Limitations

An important strength of this study is our use of qualitative methods to engage more deeply with end users. Existing stakeholder-engaged research mechanisms have allowed for patient-centered discussions regarding their needs and expectations around their health care [22]. However, youth, and particularly TGD youth, are rarely included in these conversations [23]. Thus, engaging with youth in this way represents an important first step toward developing more youth-friendly health information technology, which will not only make these services more responsive to the unique needs of youth but will likely also increase patient satisfaction and engagement with health care [23-26]. Given the current generation’s access to and familiarity with technology [27], such adolescent-informed platforms may also have additional benefits as they relate to promoting adolescent autonomy over their health care and facilitating their successful transition to adult care [28-30].

A critical limitation of our study is sample size, as the experiences of this small group of youth cannot be interpreted as representative of the broader population of TGD youth. Similarly, rates of perceived parental support among participants in our sample were quite high, which can be attributed to our recruitment strategy that focused on TGD youth who had already completed a telemedicine visit at the Seattle Children’s Gender Clinic. Care must therefore be taken not to generalize these findings to youth who may have lower rates of perceived parental support. Our study is also limited by a relative lack of diversity, which is not uncommon in clinic-based studies of TGD youth, [30] but reflects the characteristics of populations with greater access to gender-affirming care services [31]. That said, it is critically important to recognize how intersectional identities and experiences—such as those who identify as gender diverse and are Black, Indigenous, and people of color or live rurally—can further exacerbate barriers to care [31]. Furthermore, additional barriers that may disproportionately impact TGD youth, such as parent or caregiver dependence for access to care and consent to services, should also be considered when developing pediatric gender-affirming care services [6,32].

Relatedly, since we interviewed youth via Zoom and only interviewed those who had participated in a telemedicine visit, we did not hear the perspectives of youth who experience barriers to accessing these services. Specifically, those who live in areas where internet access is unavailable or less reliable may still have disproportionately less access to health care and other services. Despite the often-cited advantage of increasing access to health services among those living in medically underserved areas, we must remain aware of the fact that the rapid implementation of telemedicine could exacerbate existing disparities [33].

### Conclusions

Overall, our study supports the provision of both in-person and telemedicine modalities as options for pediatric gender-affirming care. Moreover, the benefits and drawbacks identified by TGD youth in this study can be used to inform new and developing telemedicine programs for adolescent health care as well as future work focused on building adolescent-friendly and responsive health systems.

## Abbreviations

REDCap: Research Electronic Data Capture
TGD: transgender and gender diverse
